# Endophytic fungal diversity isolated from different agro-ecosystem of Enset (*Ensete ventericosum)* in Gedeo zone, SNNPRS, Ethiopia

**DOI:** 10.1186/s12866-019-1547-y

**Published:** 2019-07-29

**Authors:** Nitin M. Chauhan, Abdissa D. Gutama, Afras Aysa

**Affiliations:** Assistant Professor of Biotechnology, College of Natural and Computational Sciences, Dilla University, 419 Dilla, Ethiopia

**Keywords:** Enset, Endophytic Fungi, Agro-ecosystem, Ethiopia, Enset leaves

## Abstract

**Background:**

Endophytic Fungi (EF) are an underexplored group of microorganisms as only a few plants have been studied with regards to their community. Diversity of EF found in young and old leaves of Enset plant has not been well studied. We analyzed and compared the colonization frequency (CF), richness diversity and fungal communities of the EF inhabiting the young and old leaves of Enset plant from Southern region of Ethiopia. Standard methods were used for isolation and identification of endophytic species from Enset leaves.

**Results:**

The study investigates the difference in quantity, in variety, in consistent pattern of community of EF along with different Enset varieties. A total number of 18 samples were analyzed and 108 morphospecies of EF were isolated and distributed among 17 genera. *Aspergillus* sp. and *Penicillium* sp. were the most common fungi reported in Enset plant. The largest numbers of EF isolates were observed in Maziya and Arkiya variety and the diversity index and species richness were found to be significant in Enset plant among these varieties. A high number of EF was isolated from old leaves in comparison to young leaves among all the varieties studied. Composition of EF at different altitudinal location also varied within each sites.

**Conclusion:**

Isolation, characterization and distribution of the EF from Enset plant is the first approach that has been conducted in the developing country like Ethiopia. The findings of the present study show that the Enset agro-forestry system produces potential variability in the colonization, richness diversity and composition of EF in Enset plants. The assemblage of EF in healthy tissues of Enset plants may indicate that some of the fungi are possible latent pathogens and some may become saprophytic.

**Electronic supplementary material:**

The online version of this article (10.1186/s12866-019-1547-y) contains supplementary material, which is available to authorized users.

## Background

Enset (*Ensete ventricosum*) is a perpetual, herbaceous and mono-carpous crop belonging to the family musaceae. The plant belongs to the monocotyledonous family comprising 37 species of *Musa* and seven species of Enset. Most fungal disease research has focused on the commercial cultivars. Enset is an orphan species and the crop is mostly cultivated for food purposes in southern part of Ethiopia [1, 2]. The edible part of Enset i.e. corm and pseudo stem provide a highly carbohydrate-rich staple or co-staple food, that feed the one fourth of Ethiopian population from south and south-western part of Ethiopia. The crop is known to tolerate extended drought periods, flooding and various diseases. Because of its drought tolerance, Enset is regarded as a priority crop in Ethiopia, where it makes a major contribution to the food safety of the country. Enset is used as staple food and is usually less influenced by the persistent drought periods that occur in Ethiopia. Comestible parts of Enset plant are pulverized and fermented into a starch-rich product called kocho. Kocho is mainly consumed after making pancake-like bread. The corm can be harvested at almost any stage of the crop, and cooked and consumed in the same way with other root and tuber crops, relieving hunger during periods of critical food shortages. Kocho can be stored for a long time (for 10 years and even more) without being spoiled [3].

Enset crop is suitable for sustainable agricultural systems due to its contribution to soil fertility. Reducing soil fertility is one of the major constraints causing yield reduction in Ethiopia. However, Enset has been harvested so intensively during droughts that some important clones have become extinct; there by reducing the genetic diversity of the crop. The crop represents 65% of the total crop production from southern regions of Ethiopia. *Coffea arabica* which is known to be originated in Ethiopia is mainly cultivated under the shade of Enset plants in Gedeo zone of southern Ethiopia, in order to achieve more yields of coffee beans and to protect the plants from environmental stress [3]. Productivity is very high compared to other crops but varies depending on edaphic factors, altitude, cultural practices and varietal differences. An integrated and comprehensive study of the biological, agricultural, ecological, social, and economic components that make up Enset based agricultural systems is needed in order to boost productivity and permit the distribution of Enset products to non- Enset growing regions of Ethiopia [4].

Endophytic microorganisms are those which inhabit the internal part of plants, causing apparently no visible changes to their hosts. They play specific roles for plants like, protecting the host-plants against insects and diseases. Some endophytic microorganisms can produce valuable pharmaceutical substances of biotechnological interest [5]. This includes symptomless latent pathogens and those fungi which also have an epiphytic stances phase of their life cycle. These microorganisms (mostly fungi and bacteria) mainly reside in host plant for all or part of their life cycle [6]. Endophytes are ubiquitous in the plant world, no report of a plant species not associated to them is known. In addition, in a given plant species individuals without endophytes are rare. Endophytes are known to affect the interactions of plants with their environment and to alter the course of their interactions with plant pathogens. In addition to represent a source of organisms for disease control and plant improvement, the study of endophytes may have an important influence in the conceptual framework where plant-pathogen interactions are interpreted and investigated [7, 8].

The association between plants and endophytes has been long-established (> 400 million years); Krings et al.*,* [9] found evidence of colonization by three different endophytes in the ancient Rhynie chert plant (*Nothia aphylla)* in Scotland. Extensive research has demonstrated that fungal endophytes are universal throughout all ecosystems and plant species [10]. On a large scale, endophytic diversity and occurrence are dependent upon biogeography; Arnold and Lutzoni [11] not only found diversity to be greater at the equator than at the poles apparently also observed occurrence to be distinct among bio-geographic regions (i.e., arctic, temperate or tropical). At a more local scale, environmental conditions such as water availability, temperature, agro-ecosystems and plant chemicals can influence diversity [12]. Also co-evolution with a host may affect endophytic presence and diversity.

Over time, some pathogenic fungi evolve to form benign or mutalistic associations with their host. Benefits such as herb ivory defense or enhanced competitive abilities [13, 14] afforded to a host may result in increased dominance of a specific endophyte conferring those benefits. Furthermore, some endophytic species (e.g., *Neotyphodium*) that confer benefits to a host can also limit colonization of other endophytes via mechanisms such as chemical production. Host genotype can affect endophyte richness, diversity and composition [15]. Endophyte communities within spotted knapweed from its native and introduced ranges differed significantly; a specific haplotype of *Alternaria alternata* was dominant in plants from the native range while *Alternaria tenuissima, Cladosporium herbarum* and an *Epicoccum* sp. were the most relatively abundant endophyte. Studies on EF community residing in banana plants were extensively studied by various authors [16–19]. However, role of EF from different agro-ecosystems in Enset (false banana) is yet to be revealed in details.

Therefore, the aim of the proposed study is to investigate whether Enset agro-ecosystem from Gedeo zone exhibit EF diversity and endophytic community associated within this area. To accomplish this task we have performed richness diversity, colonization rate of EF, fungal community resides in *Ensete ventricosum* leaves collected from different regions. This work is the primary approach to determine the EF diversity in the Enset for the first time. The results of the proposed work is highlighted and discussed below.

## Method

### Description of the study area

Gedeo zone is situated in South Nation Nationality and People Regional State (SNNPRS) of Ethiopia. The Gedeo zone is located at a distance of 369 km from Addis Ababa on Addis Ababa-Moyale international highway towards Kenya and 90 km from Hawassa, the capital city of SNNPRS. Geographically, the Zone is located North of Equator from 5° 53′N to 6° 27′N Latitude and from 38° 8′E to 38° 30′E, Longitude (see Additional file 2: Figure S1). The altitude ranges from 1500 to 3000 m and above. The zone has mean annual rainfall of 1500 mm with the mean annual temperature of 21.5 °C. The samples were taken from three distinct agro-ecological sites of Gedeo zone namely Dega, Weina-dega and Kefil-kola with an altitude ranges from 2300 to 3300, 1800–2300 and 1500–1800 masl respectively (see Additional file 1: Table S1).

### Sampling strategy and collection of sample

Three different varieties of healthy Enset plants were selected from three different sites and the sample collection procedure was based on stratification with random sampling methods. No ethical consent is required to collect the samples from studied sites. The plants were identified at genus and species level by taxonomist via leaf morphology. Sample collection was conducted in first week of December, 2016. The leaves samples were collected within the gap of 4 m each among leaves from three different varieties i.e. Maziya, Boza and Arkiya. Individual plant (*N* = 1 per variety from different agro-ecological sites) was used as replicate. One young and one old leaves were collected from each different varieties. A total number of 18 leaves samples were collected for the proposed study i.e. 2 leaves (one old and one young leaf) from three different varieties collected from each sites of Gedeo zone. The samples were totally healthy, free from any injury, fresh, green and symptomless. These sampled leaves were collected in closed sterile polythene bag and labelled, properly stored in ice bag and brought to the laboratory, then processed within 24 h, followed by surface sterilization [20].

### Sample sterilization and inoculation

Leaves samples were collected from three different varieties of healthy Enset plants from three different agro-ecological sites and leaves were thoroughly washed under running tap water before processing and followed by sequential steps: leaves of three Enset varieties samples were surface sterilized by sequentially dipping into 70% ethanol for 1 min, 1% sodium hypochlorite for 3 min and 70% ethanol for 1 min and were rinsed with sterile distilled water thoroughly to dilute or remove the chemicals, then allowed to surface dry under biological safety hood [21]. The segments of sample leaves were cut in to 2 mm length i.e. one from the base, one from the middle (right to left) and one from the apex of lamina of each leaf with the help of sterile surgical blade. Each surface-sterilized fragment of leaves was inoculated aseptically in Petri dish incorporated with Potato Dextrose Agar.

### Isolation of EF

The following fungal growth media were used to germinate fungal mycelium and sporulate. The tips of hyphae from different fungi emerging from the same leaf fragment were subculture on all of the following media; Czapek agar, Oatmeal Agar (OA), Malt Extract Agar (MEA), Corn Meal Agar (CMA), Potato Carrot Agar (PCA) were used for initiating spore formation. Chloramphenicol (50 mg/ L) was supplemented to each medium and was used to inhibit the growth of gram positive and gram negative bacterial on fungal culture. Plates containing different media lacking Enset leaves as well as surface sterilized uncut leaves sample were used as control to see any contamination. All the chemicals used were of analytical grade and were purchased from High Media Ltd., Mumbai, India.

### Morphological identification of EF

The fungi were first identified grown on potato dextrose agar (PDA) plates incubated at 25 °C for 24 to 96 h or until there was visible mycelium growth from the leaves tissue of Enset plant. Based on morphological, structural and physical nature, sub-culturing of the colonies was done by transferring single isolated colony from PDA plates to the tube containing PDA medium for identification purpose [21]. The EF that did not sporulate on PDA, they were grown separately on OA, CMA, PCA and MEA to promote sporulation.

For effective sporulation the cultures were kept in an incubator with no light for 2 weeks and later moved to 12 h fluorescent light/darkness incubator. After incubation all the fungal isolates prepared from cultures were mounted and stained with methylene blue and bromothymol blue reagent and examined with a bright-field microscope (Olympus BH-2). For characterization of the morphology of fungal isolates and identification of morpho species, the following seven morphological characteristics were used as criteria to separate and categorize. Identification was based on morphological uniqueness such as growth pattern, color of colony and medium, margin character, surface texture, colony appearance, mycelium color and structure, type of anamorph, conidiomata, conidia and conidiophore morphology (size, color, shape, ornamentation, etc.), conidiogenous cells and characteristics of the spore. [22–24].

### Statistics analysis of data

All the results obtained were analyzed by using Tukey’s pairwise comparison one way ANOVA where, *P* < 0.05 was considered statistically significant in PAST version 3.11 software [25]. The dominant fungi were defined as number of foliar EF isolates collected from each samples divided by total number of fragments incubated.

The relative frequency of colonization {% Colonization Frequency (%CF)} was estimated as the number of isolates of each species from each segment divided by total number of segments plated multiplied by 100. The percentage of total number of each isolates was determined as number of each species isolated divided by total number of endophytes multiplied by 100. The similarity between fungal communities was estimate by using Sorensens Index [26, 27].

The Shannon, Margalef diversity index and pielov evenness index were also calculated for each system. The Shannon diversity index (H′) was derived according to following equation: - ∑ (Pi ln [Pi]); where Pi = ni/N, ni = number of individuals of the species i, and *N* = total number of individuals of all species. Margalef index was calculated as: D = S - 1/LogN, where S is the number of species and N is the total number of specimens in the sample. Pielou evenness result was obtained by following equation: J’ = H′/Log (S), where H′ is the value calculated by the Shannon index and S is species richness. Evenness expresses how evenly the individual in the community are distributed among each other species. Species richness (S) is the easiest measure of biodiversity. These indices consider relative abundance of taxa. Comparison of dissimilarity percentages among EF isolated from various varieties and different sites were calculated by using by using Index of Dissimilarity (D). Diversity indices were determined by using the PAST software version 3.11 [25].

## Results

### Abundance and diversity of EF

The total numbers of 108 EF were isolated from 18 *Ensete ventericosum* leaves collected from three different varieties of Enset from three agro-ecological sites. 108 isolated EF were assigned to 17 morpho-genera which are highlighted in Table 1. Control plates did not show any growth pattern of fungi in all the media used throughout the experiment. The species identification was first carried out according to colony or hyphae morphology of the fungal cultures and spores (see Additional file 3: Figure S2). Some of the fungal isolates were identified to genus level. Among them *Aspergillus* and *Penicillium* were common. Especially, *Aspergillus* sp. with 24 EF isolates (22.22% CF) followed by *Penicillium* sp. with 11 isolates (10.18% CF) were commonly identified. The remaining 38 species of EF (67.6% CF) that were isolated from Enset leaves includes *Fusarium, Cladosporium* sp., *Epicocum nigrum,* etc. which exhibited a low abundance (≤ 2%) (Table 1). Based on these features 108 endophytes were classified into 26 morphological species or 17 morpho-genera (Table 1).

**Table 1:** Morphogenera of EF isolated from different Enset leaflets of Gedeo zone, Ethiopia and there relative frequencies

Endophytic fungi	Enset Varieties from Gedeo Zone			*N*	CF %
	Maziya	Boza	Arkiya		
*Alternaria alternata*	2	3	4	9	8.33
*Aspergillus niger*	2	2	2	6	5.56
*Chaetomium glubosum*	2	2	2	6	5.56
*Colletotrichum coffeanum*	2	1	3	6	5.56
*Epicoccum nigrum*	3	3	0	6	5.56
*Aspergillus bisporus*	1	2	2	5	4.63
*Aspergillus flavus*	1	2	2	5	4.63
*Aureobasidium pullulans*	2	1	2	5	4.63
*Byssochlamys nivea*	2	1	2	5	4.63
*Penicillium radicum*	2	2	1	5	4.63
*Scopulariopsis brevicaulis*	1	1	3	5	4.63
*Aspergillus japanicus*	1	2	1	4	3.70
*Eurotium amstelodami*	1	2	1	4	3.70
*Pestalotiopsis maculans*	1	2	1	4	3.70
*Verticillium nivea*	1	2	1	4	3.70
*Aspergillus oryzae*	2	1	0	3	2.78
*Boehravia diffusa*	1	2	0	3	2.78
*Fusarium* sp*.*	1	1	1	3	2.78
*Getricum* sp*.*	0	1	2	3	2.78
*Phoma exigua*	2	1	0	3	2.78
*Penicillium coffeae*	2	1	0	3	2.78
*Penicillium corloyhim*	1	1	1	3	2.78
*Acremonium murorum*	0	2	0	2	1.85
*Acermonium* sp.	0	2	0	2	1.85
*Aspergillus* sp.	0	1	0	1	0.93
Total	**33**	**42**	**33**	**108**	**100.00**

^*^N = Total number, *CF*=Colonization Frequency

### Species richness distribution of EF among three varieties

Some species of EF isolated were specific to only one or two Enset varieties studied. For instance, *Aspergillus* sp., *Acermonium* sp., *Acremonium murorum* were isolated only from Boza variety. Whereas, *Epicoccum nigrum*, *Phoma exigua*, *Boehravia diffusa*, *Aspergillus oryzae* and *Penicillium coffeae* were found in both Maziya and Boza Enset varieties. On the other hand, *Penicillium corloyhim*, *Pestalotiopsis maculans*, *Verticillium nivea*, *Aspergillus bisporus*, *Aspergillus flavus*, *Alternaria alternata*, *Aureobasidium pullulans*, *Aspergillus japanicus*, *Colletotrichum coffeanum*, *Scopulariopsis brevicaulis*, *Byssochlamys nivea*, *Chaetomium glubosum*, *Penicillium radicum*, *Aspergillus niger*, *Fusarium* sp. and *Eurotium amstelodami* were assemblage in almost all varieties of Enset plants studied (Table 1).

### Morphospecies distributions of EF among three Enset varieties

Numbers of EF in different varieties of Enset plants did not have any significance difference among each other (*P* = 0.43). Maziya variety was found to be dominant over Boza and Arkiya varieties with a recorded CF of 37.02%. Whereas, Boza and Arkiya varieties produced 30.54 and 32.39% of EF respectively. When comparing the varieties of Enset plants at different agro-ecology sites, 23 EF were isolated from Dega sites than that of Weina-dega and Kefil-kola sites where, 16 and 01 EF were recorded respectively in Maziya variety. Similar results were recorded for Boza variety where, 20, 12 and 1 species of EF were isolated from Dega, Weina-dega and Kefil-kola sites respectively. However in Arkiya variety, Weina-dega site has more EF i.e. 18 than that of Dega and Kefil-kola where, 10 and 7 EF were observed (Table 2).

**Table 2:** Diversity of EF isolated from three different agro-ecological sites in three different varieties of *Ensete ventericosum*

Enset varieties	Agro-ecology Sites	No. of E. F	CF %	Total CF %
Maziya	Dega	23	21.29	37.02
	W. dega	16	14.8	
	Kolla	1	0.92	
Boza	Dega	20	18.51	30.54
	W. dega	12	11.11	
	Kolla	1	0.92	
Arkiya	Dega	10	9.25	32.39
	W. dega	18	16.66	
	Kolla	7	6.48	
Total		108	100	100

### Diversity and distribution of EF among different agro-ecology sites

**Fig. 1:**
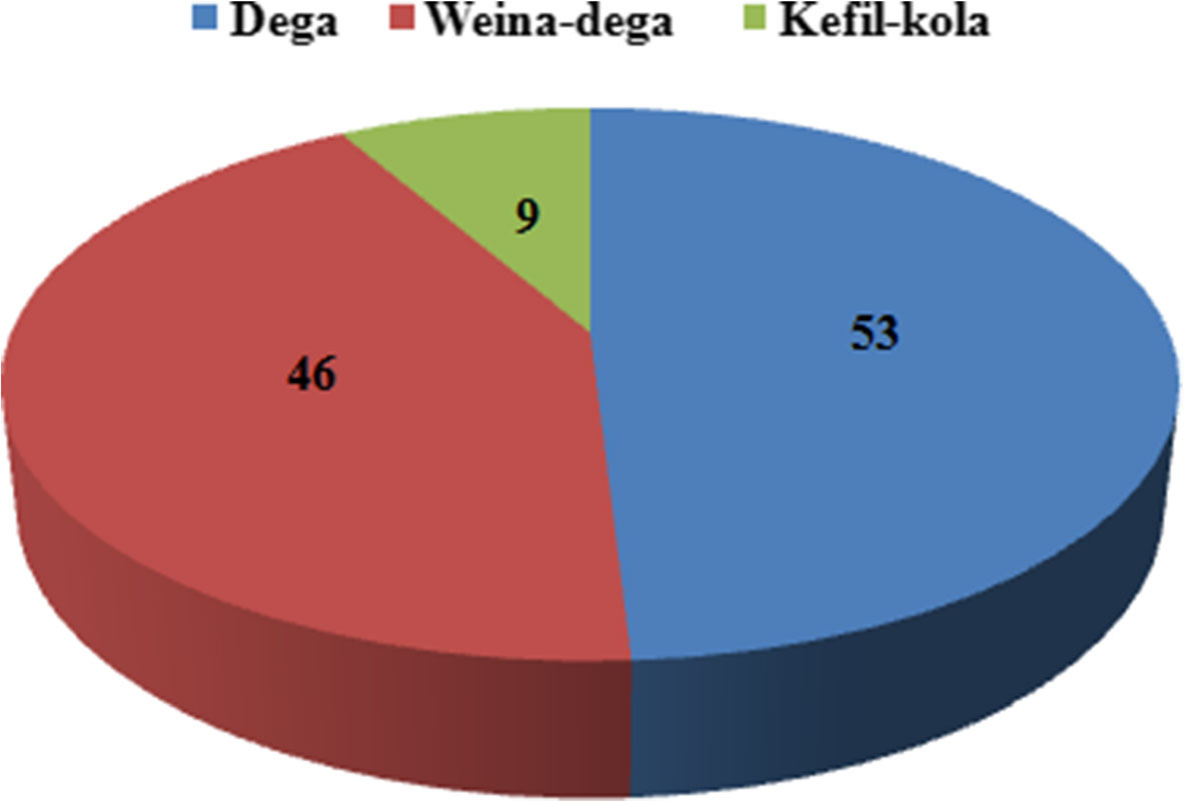
Diversity and distribution of EF in Enset plant collected from different agro-ecology sites of Gedeo zone, Ethopia. Values represent the number of EF isolated from different agro-ecological sites. The segment of sample leaves collected from different sites were placed aseptically on various media and incubated at 25 °C for 24–96 h. EF was identified on the basis of seven morphological characteristics after incubation

Among the different agro-ecological areas studied, various species of common EF were isolated. According to different altitudinal location the abundances of endophytic fungal species also varied within each sites. The high altitude site i.e. Dega recorded 53 (49.07%) species of EF followed by mid altitude site i.e. Weina-dega reported 46 (42.57%) species of EF. However, 9 (8.33%) EF species were isolated from low altitude site namely Kefil-kola (Fig. 1).

### Diversity richness comparison among three different varieties of Enset

Some of the results according to these parameters were significantly similar to each other. For example, the comparison of Maziya Dega (MD) with Maziya Kolla (MK) and Boza Kolla (BK) showed 0.0121 and 0.031 similarity respectively. While, comparisons of MK with Boza Dega (BD), Boza Weina-dega (BW) and Arkiya Weina-dega (AW) produced 0.0063, 0.034 and 0.015 similarity respectively. But majority of the results according to these parameters were significantly dissimilar with large groups. Likewise the comparison of Arkiya Kolla (AK) with MD, Maziya Weina-dega (MW), BD, Arkiya Dega (AD) and AW resulted in 3.50, 0.70, 2.10, 0.70 and 1.75% of dissimilarity respectively. The other comparison such as BK with MD, MK, BD, AD and AK reported 6.30, 0.35, 4.90, 0.862 and 0.55% of dissimilarity. Highest percentage of dissimilarity i.e. 6.230 was noted among comparison of BK with MD. And lowest dissimilarity i.e. 0.0001 was seen among MD and MK (Table 3).

**Table 3:** Comparison of dissimilarity percentage of EF among three varieties of Enset plants collected from three different sites

	MD	MW	MK	BD	BW	BK	AD	AW	AK
MD	0	0.55	**0.0001**	0.98	0.86	**0.00031**	0.07	0.9	0.2
MW	2.8	0	0.13	0.9	0.9	0.24	0.98	0.9	0.9
MK	6.6	3.8	0	**0.006**	**0.034**	1	0.72	**0.015**	0.38
BD	1.4	1.4	5.2	0	0.9	**0.015**	0.55	1	0.86
BW	2.10	0.70	4.5	0.70	0	**0.07**	0.86	1	0.98
BK	6.30	3.50	0.35	4.9	4.20	0	0.86	**0.034**	0.55
AD	4.2	1.4	2.45	2.80	2.10	2.10	0	0.72	0.99
AW	1.75	1.05	4.9	0.35	0.35	4.5	2	0	0.94
AK	3.5	0.70	3.15	2.10	1.40	2.80	0.70	1.75	0

A = Arkiya B = Boza D = Dega K=Kolla M = Maziya W = Weina-dega. Values represented are dissimilarity percentage calculated by using Index of Dissimilarity (D). Bold values represent some percentage of similarities among Enset plants varieties collected from different sites

### Distribution of EF among young and old leaves of Enset

**Fig. 2:**
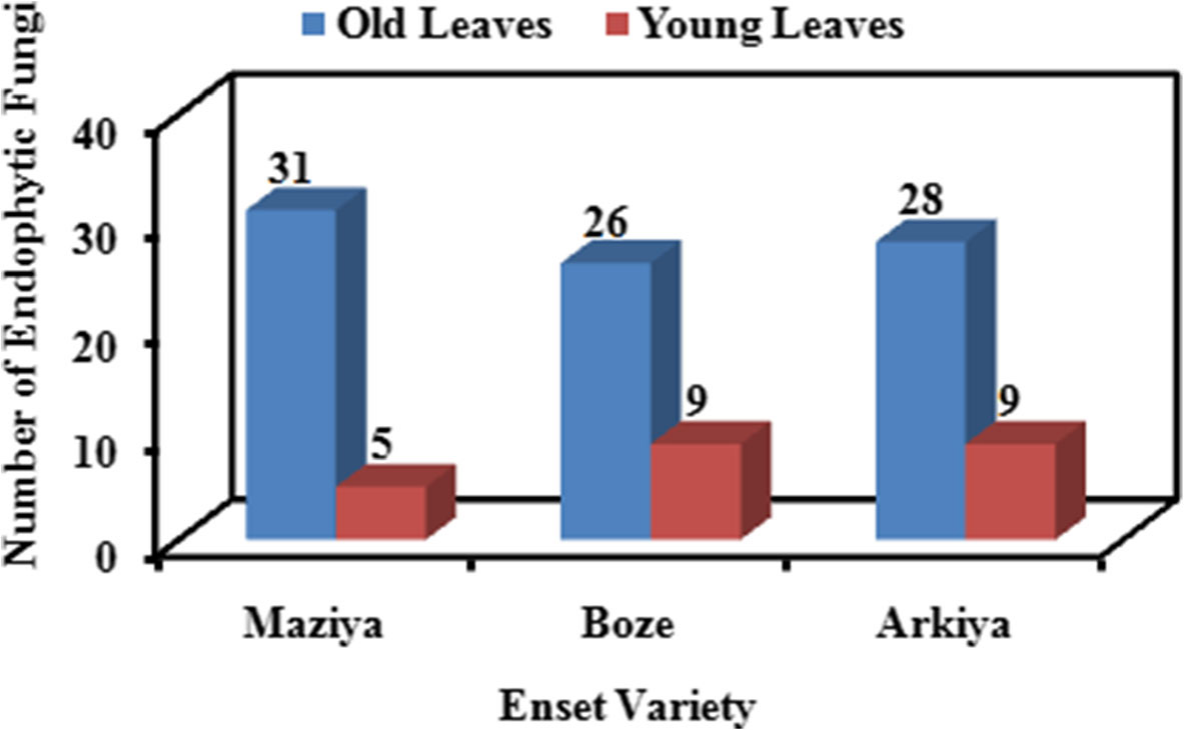
Distribution of EF among young and old leaves of Enset plants accumulated from Gedeo zone, Ethiopia. The segment of old and young Enset leaves were placed aseptically on various media and incubated at 25 °C for 24–96 h. EF was identified on the basis of seven morphological characteristics after incubation

The composition of EF was significantly varied among young and old leaves of Enset (*P* = 0.0004). A high number of EF was isolated from old leaves in comparison to young leaves. For example, 85 EF species were recorded in old leaves whereas, 23 species of EF were seen in young leaves of Enset. Among the different varieties of Enset plants, 31, 26 and 28 different species of EF were isolated from old leaves of Maziya, Boza and Arkiya respectively. On the other 5, 9 and 9 EF species were recorded in young leaves of Maziya, Boza and Arkiya varieties respectively (Fig. 2). Table 4 highlights Shannon’s diversity index (H′), Simpson diversity (Simpson_1-D), Dominance index (Dominanac_D), Evenness_e^H/S and other diversity indices of EF isolated from young and old leaves. Highest biodiversity of EF was observed in old leaves (Shannon diversity = 1.096, Evenness_e^H/S = 0.9974, Simpson diversity = 0.6649) followed by young leaves (Shannon diversity = 1.066, Evenness_e^H/S = 0.968, Simpson diversity = 0.6465). However, Dominance index, Margaleaf, Berger-Parker and Fisher-alpha constant were found to be highest for young leaf when compare to old leaf.

**Table 4:** Diversity indices of EF isolated from old and young leaves of Enset

Index	Old leaves	Young leaves
Taxa_S	3	3
Individuals	85	23
Dominance_D	0.3351	0.3535
Simpson_1-D	0.6649	0.6465
Shannon_H	1.096	1.066
Evenness_e^H/S	0.9974	0.968
Brillouin	1.041	0.9224
Menhinick	0.3254	0.6255
Margalef	0.4502	0.6379
Equitability_J	0.9976	0.9704
Fisher_alpha	0.606	0.9211
Berger-Parker	03647	0.3913
Chao-1	3	3

## Discussion

The present study provided valuable data regarding biodiversity and impact of determinant factors on composition of fungal endophyte communities that attribute symbiotic micro-flora of widely used Enset plants in the proposed region. It has been demonstrated that EF can profoundly influence different aspects of plant patho-physiology and actively participate in bio-mechanisms by which plant growth and regeneration is controlled. Thus, understanding the structure of endophyte assemblages is required for further approaches in order to improve the agro-economic status of the Enset production and to find novel applications of these microorganisms and their metabolites in horticultural, or even for medicinal purpose. Results achieved by the present study were applied for an analytical assessment to explicate the composition of EF assemblages on Enset plants in Ethiopia. Biodiversity of EF associated with host plant and structure of endophyte communities are dynamically convertible depending on plant physiology, environmental stresses, their interplay with other parasitic or pathogenic microorganisms and bio-geographical factors [10]. Endophytes also tend to be host-specific that can change the prevalence of endophytic taxa in a particular plant genus between different species. As well, divergent endophyte composition may be harbored in host organs due to the histological differences and availability of nutrients by which endophyte colonization is conducted in a tissue-specific manner within a distinctive host species [10]. Along with temporal changes in the niche where both host plant and endophyte inhabit, host species and harboring tissue are consequently the most important factors modulating endophyte diversity [10]. Various classical protocols have been developed for studying any substratum or group of fungi, and are described in detail by various researchers and are easily available as published data [28, 29]. Therefore, the present study was conducted with defined methodology so that it could be applied to obtain such data and infer a comprehensive view about composition of fungal endophyte communities associated with Enset plants.

In this study, a total number of 108 EF were isolated that are assigned to 17 morpho-genera or 26 morphological species (Table 1). Total numbers of 53, 46 and 9 EF were isolated from Dega, Weina-dega and Kefil-kola respectively (Fig. 2). Results of our study is supported by Arnold et al.*,* [10], who suggest that diversity of EF strongly influenced by the traits of ecological environment and geographical location. EF are generally ubiquitous in all plant species studied and *Ensete ventericosum* plants were not known to harbor any EF. Results of our study reported that *Ensete ventericosum* plants had a huge number of endophytic fungal associations. Presence of EF reveals prevalence in plant tissues under taken for investigation. However, in this study the richness in diversity of EF was found among Enset varieties. During course of study, fungal endophytes were isolated at definite conditions using specific and general media for fungal growth. The fungal endophytes that were successfully sub-cultured were identified using morphological characterization.

Three Enset varieties were studied for the composition of EF were principally isolated from young and old leaves of each Enset varieties in the area. It was found that the number of EF isolated from Enset varieties of the area increased with the age of the plants, with 85 isolates being obtained from old leaves of three Enset varieties and 23 isolates were recorded in young leaves of three Enset varieties. This variation of number of isolates may be due to the impact of leafs age dependent production of biochemical metabolites (in amount and type) variation and nutrient composition in leaf of Enset varieties of the area. Since, production of biochemical metabolites (in amount and type) variation and nutrient composition directly or indirectly govern existence and survival of EF the host plant. For example, genera of isolates like *Aspergillus* sp*.* and *Penicillium* sp*.* were totally depend on aged leaf of Enset plants. This could have been due to a lack of essential and insufficient nutrients were reported for EF by Pimentel et al.*,* [30] showed that some of the essential nutrients needed by such fungi and in sufficient amount were unavailable during the young stage of Soybean leaves. Similar result was obtained in our study where, *Aspergillus* sp. and *Penicillium* sp*.* were recorded only from old leaves and were totally absent in young leaves (results not shown).

Knowledge and awareness of diversity of foliar EF in Enset leaves have significant advantage for identification of phytopathogenic fungi that might live as endophytes during part of their life cycle [31]. Abundance of foliar EF in old leaves of the three Enset varieties was varied than those of their young leaves of their own varieties (85 from old and 23 from young leaves were found respectively). This result are in the line of the study of Arnold and Herre, [8] who reported that age of the leaves increase from young to mature diversity and richness of endophytic mycobiota increases within the plant. Because mature leaves may have supported higher endophytic abundance may be, due to their higher biomass providing more sites and resources for colonization when compared to young leaves. Likewise leaf exposure time may also have accounted for increased density of EF due to horizontally transition and accumulated over leaf lifetimes.

Arnold and Herre, [8] were also found that the number of isolates decreased with increasing age of the plants. One of several possibilities were explained by Garcia et al.*,* [32] are since pruned height of young trees is lower, so that the first branch will be closer to the reservoir of inocula present in the previous year’s litter, and therefore fungi may spread relatively rapidly towards the upper third of the canopy. The other reason could be due to a lack of essential nutrients; those essential nutrients needed by EF were unavailable during the maturation and senescence of plants [33]. Such kind of phenomenon was not observed in our proposed study. Possible reason may be young Enset leaves may not produced essential nutrients that have potential to support the growth of certain EF. As such, high number of EF was isolated from old leaves in comparison to young Enset leaves.

According to the report variation of their diversity could be obtained by abiotic variations on the sites of host plants which govern differences in abiotic factors or local sources of spores. The other factor could be interactions between the endophytes themselves. It is known that endophytes produce an array of metabolites that are active against other microbes. For example, an endophytic fungus inhibits growth of pathogenic fungi within the host plant. The other few species of EF inhabit and isolated from three varieties of Enset plants. For instance, *Aspergillus* sp., *Acermonium* sp. and *Acremonium murorum* were isolated only from Boza Enset variety, where as *Epicoccum nigrum, Phoma exigua, Boehravia diffusa, Aspergillus oryzae and Penicillium coffeae* were isolated from both Maziya and Boza Enset varieties and many more as highlighted in (Table 1). These groups of endophytic fungal community might be belong to the class of fungi which involved in the decomposition of plant litter, related to known saprotrophic fungi and unidentified relatives have previously been detected in soil and decaying plant material [34]. Similar results were also mentioned in the study of Persoh [34] showed that, the ‘latent decomposers’ are not randomly distributed among host plants instead they are host specific species.

Large number of dissimilarity was observed among Enset varieties and different agro-ecological sites with an exception of comparison of MD with BK and MK, MK with BD, BW and AW which has very least percentage of dissimilarity. However, majority of the result according to this parameter, we found dissimilarities in the EF colonization among varieties and different sites studied (Table 3). Those results suggest that agro-forestry system may influence the assemblage of EF of Enset plants among the varieties and different sites studied. Previous studies on the composition EF of herbaceous cultivable plants, such as cotton and maize, have shown no similarities in EF communities based on farming practice [35, 36]. However, our results are in agreement with Pancher et al., (2012) [37] who reported dissimilarity in EF colonization isolated from *Vitis vinifera* under various vineyard management practices. Similar results were also published by Saucedo-Garcia et al., [38] as they reports the dissimilarity in the composition of EF from different agro-ecosystems of *Coffea arabica* L. in two regions of Veracruz, Mexico. These indicate that the plantation management techniques in agro-forestry systems of southern Ethiopia, such as coffee plantation and Enset, might influence the assemblage of EF composition. More sampling in future will help to elucidate the factors that influence the EF colonization, but as shown in this report, some of those factors might be the geographical location and agro-forestry system of the Enset plantations has potential to assemblage of EF among Enset leaves.

A comparison of the Shannon diversity index for fungal communities of young and old leaves showed the highest diversity in the old leaves, followed by young leaves. There were significant differences in the diversity of fungal communities among different leaves of Enset (one-way ANOVA, *P* = 0.0004). The diversity indices of EF species associated with Enset leaves are summarized in Table 4. The Margalef index can reflect the richness of EF species. Result of our study showed that Margalef index was found to be highest for young leaves than that of old leaves. Larger the values of Margalef, the richer the species of EF are [39]. However, in our study no larger Margaleaf values were observed and no significant differences in the Margaleaf indices were recorded for young and old leaves. Thus, it can be suggest that no richest species of EF are present in Enset leaves. The species diversity can be analyzed by the Shannon index and Simpson diversity index. These indices are taken into account for the heterogeneity/homogeneity of the species frequencies. Generally, higher the Shannon’s diversity index (commonly ranging between 1.5 and 4.5) and the closer the Simpson’s diversity index to 1, the more intensified heritable variation and stronger adaptive capacity for micro-environmental change the EF communities as they tended to expand the distribution range and enter new environments. As such in our study no heritable variation and stronger adaptive capacity for micro-environmental change in the EF communities can be assumed as the values of Shannon’s diversity index does not range between 1.5 and 4.5 and Simpson’s diversity index was not approach to 1. The Dominance index was used to evaluate the ecological dominance of a community. If a higher Dominance index is observed in the community, it indicates that the community might have low species diversity and evenness. However, in our study no higher values of Dominance index were observed suggest that community might have high species of diversity and evenness. Given the small number of samples (9 Enset plants), it is hard to estimate the total number of endophytic diversity of each individual. More sampling from different parts of Enset growing region will help to elucidate the factors that influence the EF diversity in future.

The findings of the present study show that the Enset agro-forestry system produces potential variability in the colonization, richness diversity, and composition of EF in Enset plants. The results of the proposed studies provide an opportunity to understand the potential use of some EF as producers of relevant products in the management of different pests and pathogens of Enset as well as coffee where, coffee is cultivated under the shade of Enset plant which is widely practiced in Gedeo zone of southern Ethiopia., to discover new drugs with novel antifungal activities, and to understand the potential role of EF as leading controls of pest-population.

## Conclusion

Isolation, characterization and distribution of the EF from Enset plant is the first study that has been conducted in the developing country like Ethiopia. The result of the present study shows that the Enset agro-forestry system produces potential variability in the colonization, richness diversity and composition of EF in Enset plants. The present study also indicate that the Enset agro-forestry system produces variation in assemblage of EF in Enset plants and may enhance the composition of EF in coffee plants especially in Gedeo zone, where coffee is planted under the shade of Enset plant. Another study from our lab demonstrated that coffee plants grown under the shade of Enset plants are more colonized by EF in comparison to un-shaded coffee plants (unpublished data). Thus, Enset plants have a potential role in colonization of coffee plants and provide protection mechanism against common coffee pathogens around Gedeo zone. In addition, it demonstrated that the region in which Enset is cultivated plays a vital role that influences these parameters. The assemblage of EF in healthy tissues of Enset plants may indicate that some of the fungi are possible latent pathogens and some may become saprophytic. More studies by the use of molecular and physiological tools to identify, individually and collectively, the functional and ecological significance of EF in Enset plants under diverse ecological and geographical conditions will be very significant.

## Additional files

Additional file 1: **Table S1.** Description of study area and collection of samples (PDF 60 kb)

Additional file 2: **Figure S1.** Map of study area of Gedeo zone, Ethiopia (PDF 132 kb)

Additional file 3: **Figure S2.** Macroscopic pictures of EF isolated from *Ensete ventericosum (PDF 101 kb)*

## Data Availability

Some data generated or analysed in this study is available in this article and in Additional files 1-3. Raw data can be obtained from the corresponding author on reasonable request.

## Abbreviations

AK: Arkiya Kolla
MW: Maziya Weina-dega
AD: Arkiya Dega
AW: Arkiya Weina-dega
BD: Boza Dega
BK: Boza Kolla
BW: Boza Weina-dega
CF: Colonization Frequency
EF: Endophytic Fungi
MEA: Malt Extract Agar
CMA: Corn Meal Agar
MK: Maziya Kolla
OA: Oatmeal Agar
PCA: Potato Carrot Agar
MD: Maziya Dega
PDA: Potato Dextrose Agar
SNNPRS: South Nation Nationality and People Regional State
