# Supplementary material for: Endophytic fungal diversity isolated from different agro-ecosystem of Enset (Ensete ventericosum) in Gedeo zone, SNNPRS, Ethiopia
Source: BMC Microbiol. 2019 Jul 29;19:172. doi: 10.1186/s12866-019-1547-y (PMC6664548; doi:10.1186/s12866-019-1547-y)
Supplement: Supplementary file 1 — Table S1. Description of study area and collection of samples (PDF 60 kb) [file 12866_2019_1547_MOESM1_ESM.pdf]

Table S1: Description of study area and collection of samples

Name of the site	Enset Varieties	Type of sample collected	Total number of sample collected	Temperature (°C)	Annual Rainfall (mm)	Altitude (masl)
**Dega**	Maziya	One old and one young leaf	2	10-14	1500-2000	2300-3300
	Boza	One old and one young leaf	2			
	Arkiya	One old and one young leaf	2			
**Weina-dega**	Maziya	One old and one young leaf	2	13-18	1150-1350	1800-2300
	Boza	One old and one young leaf	2			
	Arkiya	One old and one young leaf	2			
**Kefil-kola**	Maziya	One old and one young leaf	2	15-27	750-1150	1500-1800
	Boza	One old and one young leaf	2			
	Arkiya	One old and one young leaf	2			
