# Supplementary figures and images for: Endophytic fungal diversity isolated from different agro-ecosystem of Enset (Ensete ventericosum) in Gedeo zone, SNNPRS, Ethiopia
Source: BMC Microbiol. 2019 Jul 29;19:172. doi: 10.1186/s12866-019-1547-y (PMC6664548; doi:10.1186/s12866-019-1547-y)

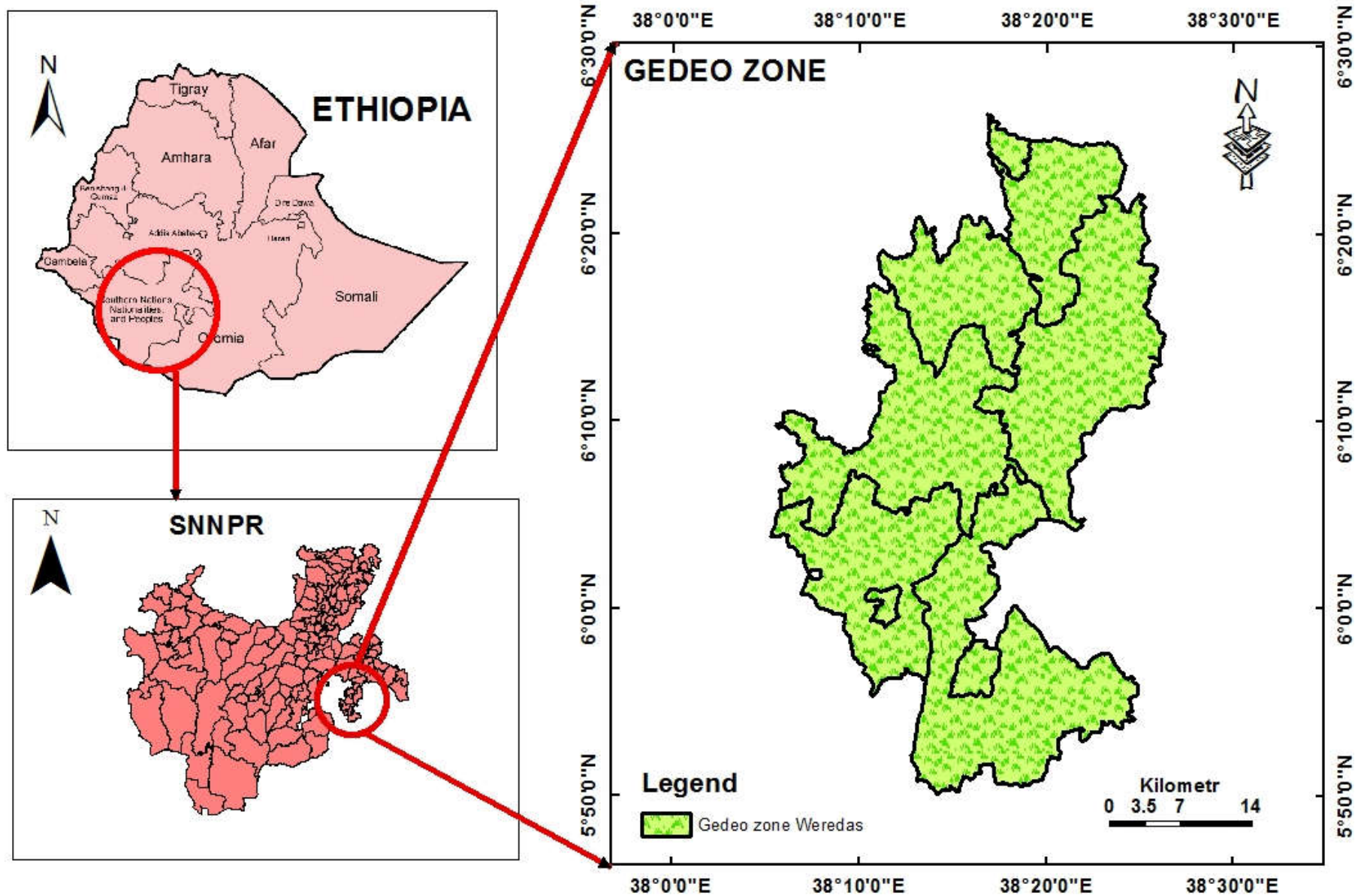

Figure S1

Supplement: Supplementary file 2 — Figure S1. Map of study area of Gedeo zone, Ethiopia (PDF 132 kb) [file 12866_2019_1547_MOESM2_ESM.pdf]

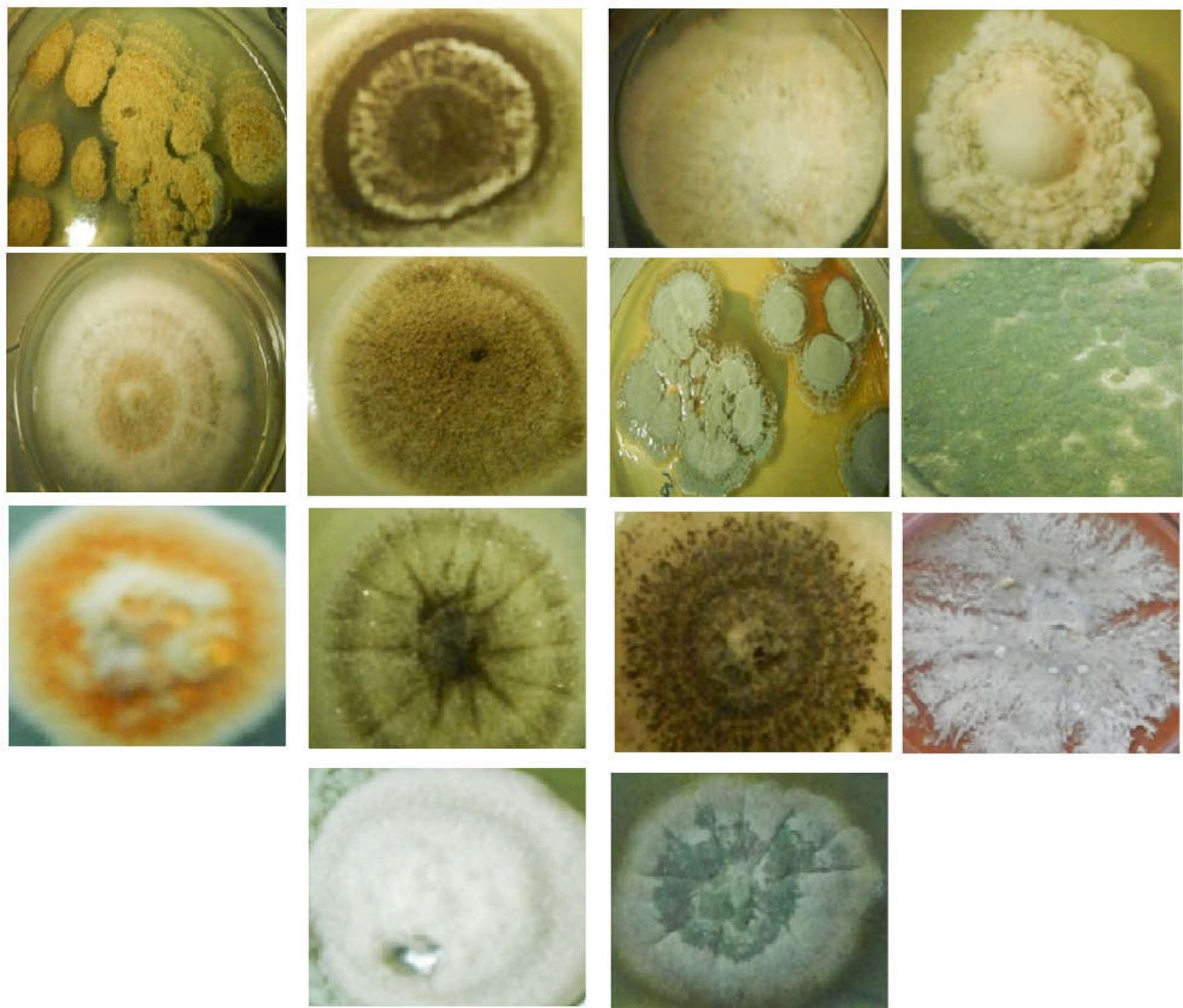

**Figure S2**

Supplement: Supplementary file 3 — Figure S2. Macroscopic pictures of EF isolated from Ensete ventericosum (PDF 101 kb) [file 12866_2019_1547_MOESM3_ESM.pdf]
